# Recent advances in liquid-metal-based wearable electronics and materials

**DOI:** 10.1016/j.isci.2021.102698

**Published:** 2021-06-09

**Authors:** Phillip Won, Seongmin Jeong, Carmel Majidi, Seung Hwan Ko

**Affiliations:** 1 Department of Mechanical Engineering, Carnegie Mellon University, Pittsburgh, PA 15213, USA; 2 Department of Mechanical Engineering, Seoul National University, Seoul 08826, Korea; 3 Institute of Advanced Machines and Design / Institute of Engineering Research, Seoul National University, Seoul 08826, Korea

**Keywords:** Bioelectronics, Metals, Electronic materials, Devices

## Abstract

Soft wearable electronics are rapidly developing through exploration of new materials, fabrication approaches, and design concepts. Although there have been many efforts for decades, a resurgence of interest in liquid metals (LMs) for sensing and wiring functional properties of materials in soft wearable electronics has brought great advances in wearable electronics and materials. Various forms of LMs enable many routes to fabricate flexible and stretchable sensors, circuits, and functional wearables with many desirable properties. This review article presents a systematic overview of recent progresses in LM-enabled wearable electronics that have been achieved through material innovations and the discovery of new fabrication approaches and design architectures. We also present applications of wearable LM technologies for physiological sensing, activity tracking, and energy harvesting. Finally, we discuss a perspective on future opportunities and challenges for wearable LM electronics as this field continues to grow.

## Graphical abstract

**Figure:**
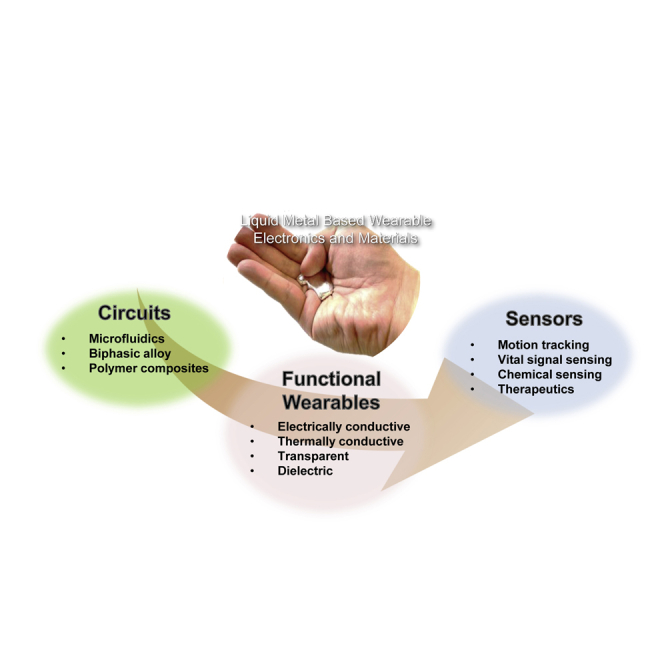

## Introduction

Stretchable wearable electronics have recently gathered significant interests to a wide range of fields owing to many findings of such electronic wearables proven to provide advantageous features for displays, healthcare monitoring, and other convenient capabilities from well-matched mechanical compliance and portability to the human body. As a consequence, more extensive works that meet with needs in more skin-like mechanical compliance, functionalities, and device performances have been developed to establish more advanced forms of daily lives with these wearable electronics. The developments of recent wearable electronics mainly involve a variety of sensors, energy-harvesting or storage devices, optoelectronics and wireless antennas (Song et al., 2020b). Although conductive materials are the core component that comprises wearable circuits or sensors, regardless of several routes to fabricate conductors flexible or stretchable through wavy pattern (Kim et al., 2011; Vachicouras et al., 2019; Yeo et al., 2013), buckled prestrain surfaces (Lee et al., 2012b; Wang et al., 2017; Xu and Zhu, 2012), and nanocomposites (Hu et al., 2016; Matsuhisa et al., 2015) still face practical challenges, considering repetitive usage, degradation of nanomaterials, and high-cost fabrication (Kim et al., 2016; Kwon et al., 2018). Yet, the introduction of eutectic gallium indium (EGaIn) or other gallium-based liquid metal (LM) alloys, which exist as liquids at room temperature (<21°C) as well as with body temperatures (36.5°C) unlike other LM alloys that are very unique owing to their combination of fluidic liquid and conductive (<3.6 x 10^−6^ S m^−1^) metallic properties, has been received considerable attentions to tackle current challenges in wearable electronics and materials (Sun et al., 2020b; Wang and Liu, 2016).

**Figure 1:**
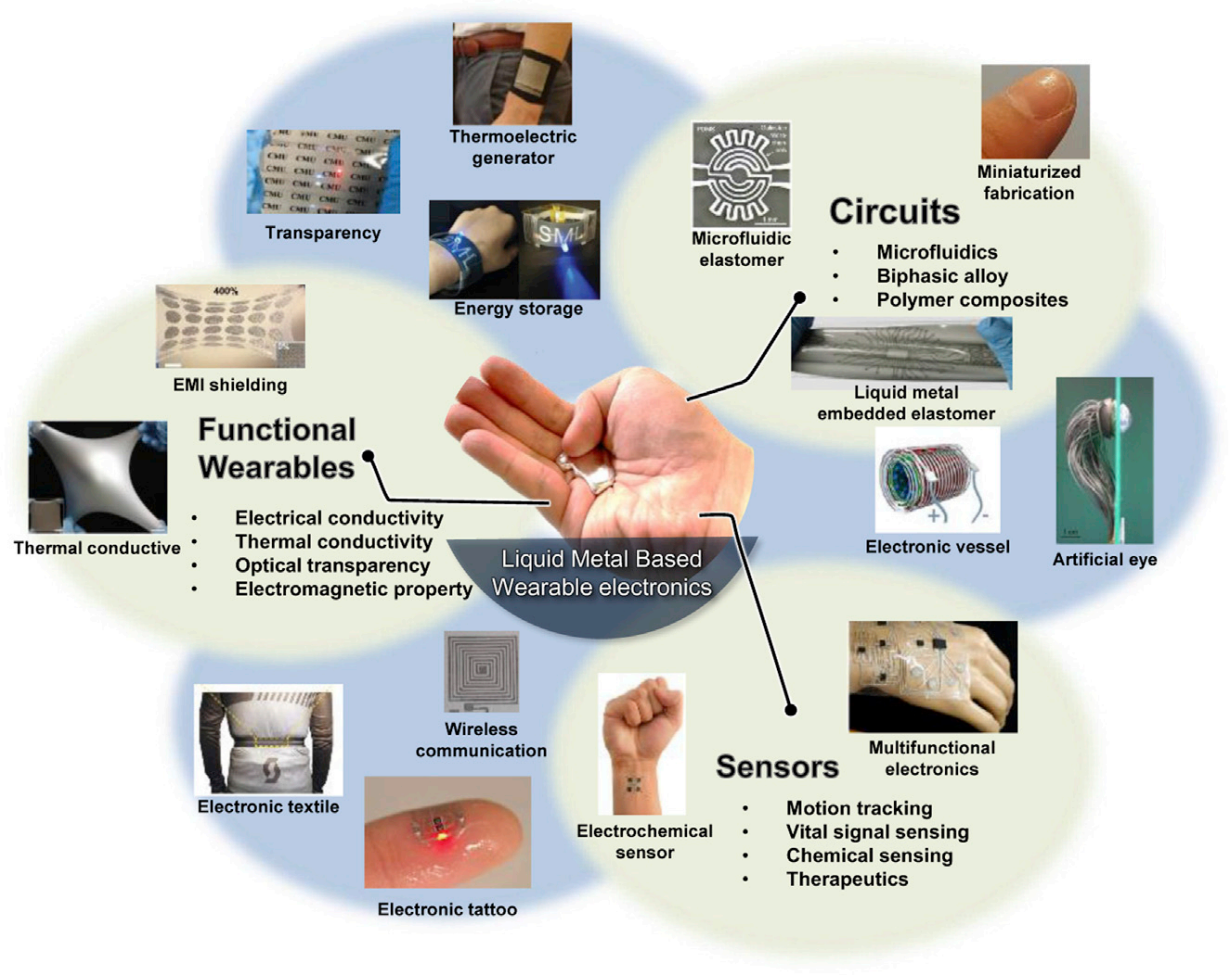
Overview of recent advances in liquid-metal-based wearable electronics Microfluidic elastomer. Reproduced with Permission (Gao et al., 2017). Copyright, Wiley-VCH. Miniaturized fabrication. Reproduced with Permission (Kim et al., 2020d). Copyright, Springer Nature. Liquid-metal-embedded elastomer. Reproduced with Permission (Markvicka et al., 2018). Copyright, Springer Nature. Electronic vessel. Reproduced with Permission (Cheng et al., 2020). Copyright, Cell Press. Artificial eye. Reproduced with Permission (Gu et al., 2020). Copyright 2020, Springer Nature. Multifunctional electronics. Reproduced with Permission (Lopes et al., 2021). Copyright, American Chemical Society. Electrochemical sensor. Reproduced with Permission (Silva et al., 2020b). Copyright, Wiley-VCH. Wireless communication. Reproduced with Permission (Alberto et al., 2020). Copyright, Springer Nature. Electronic tattoo. Reproduced with Permission (Tavakoli et al., 2018). Copyright, Wiley-VCH. Electronic textile. Reproduced with Permission (Dong et al., 2020). Copyright, Springer Nature. Thermal conductive. Reproduced with Permission (Bartlett et al., 2017). Copyright, Proceedings of National Academy of Science. EMI shielding. Reproduced with Permission (Yao et al., 2020). Copyright, Wiley-VCH. Transparency. Reproduced with Permission (Pan et al., 2018). Copyright, Wiley-VCH. Energy storage. Reproduced with Permission (Liu et al., 2019a). Copyright, Wiley-VCH. Thermoelectric generator. Reproduced with Permission (Zadan et al., 2020). Copyright 2020, American Chemical Society.

LM-based wearable sensors and materials especially exhibit very unique mechanical (Kazem et al., 2018), electrical (Fassler and Majidi, 2015), thermal (Bartlett et al., 2017), optical (Pan et al., 2018), electromagnetic (Yao et al., 2020), and dielectric(Pan et al., 2019) properties compared with other soft or conductive materials alone while maintaining mechanical compliance, making electronics more accessible to human body, clothing, and even tissues (Cheng et al., 2020; Li et al., 2020b; Pan et al., 2020; Ren et al., 2020; Wang et al., 2020). Depending on fabrication approaches, the conductors composed of the same materials composition can be changed dramatically. Comparatively, recent development of LM-based wearable electronic devices and materials is found in a broad range of applications such as wearable computing (Ozutemiz et al., 2018), health monitoring (Alberto et al., 2020; Kim et al., 2019a), biomedical therapeutics (Wang et al., 2019), human-machine interaction (Tang et al., 2021), and virtual reality(VR)/augmented reality (Oh et al., 2020). In this progress report, we aim to review recently developed the state of arts in fabrication approaches, materials properties, and creative applications for LM-based wearable electronics and materials, further improving the quality of life and society (Figure 1). The first section of this report will cover the strategies for circuit fabrication by categorizing them into three architectures to describe its advantage and challenges and also shows material properties that exhibit with LM inclusions. Furthermore, the recently reported LM-based wearable applications including various sensors, energy-harvesting/storage devices, antenna, and even implantable devices will be further explored. Finally, the summary and outlook for LM-based wearable electrics and materials for unexplored areas and emerging applications are discussed.

## Strategies for fabricating LM-based stretchable conductors

**Figure 2:**
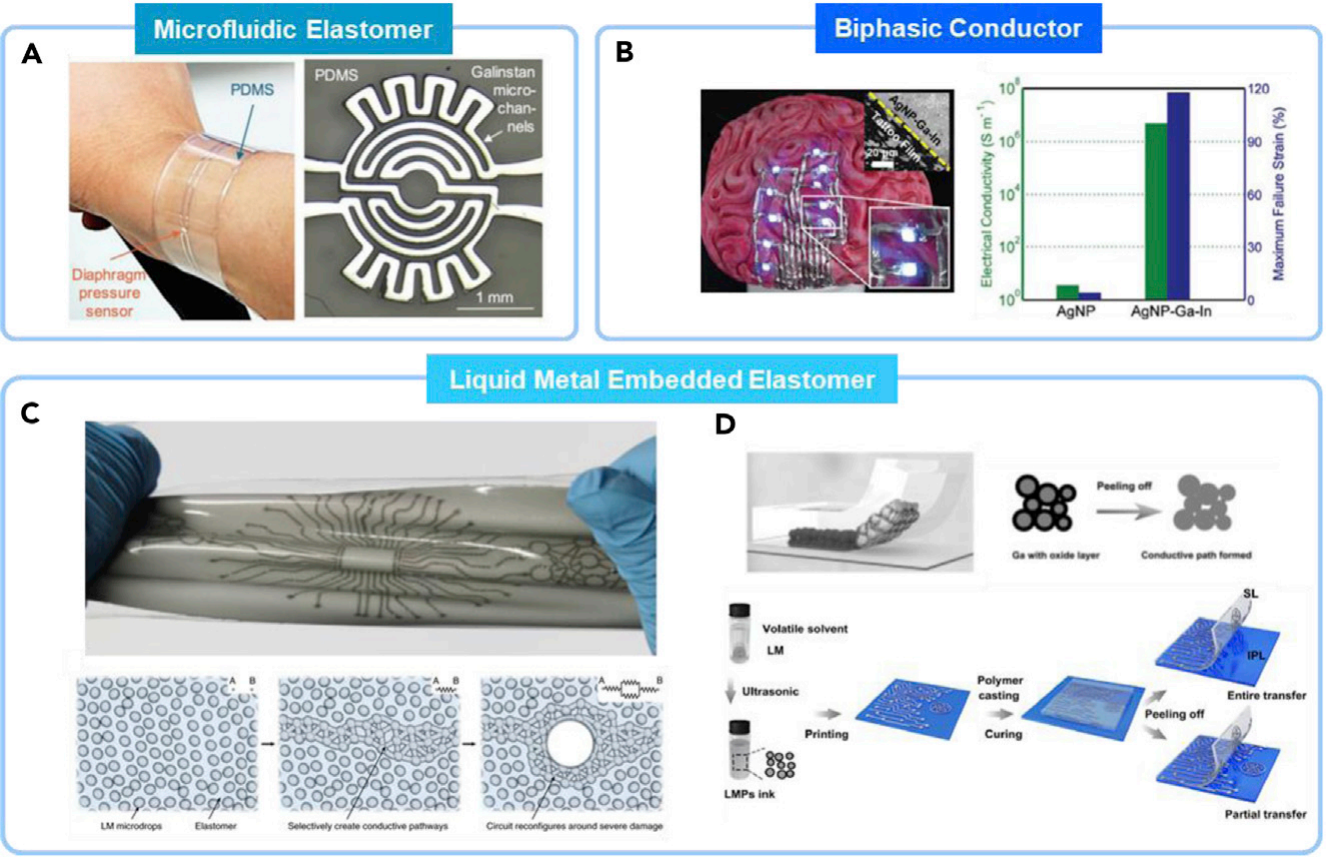
Strategies for fabrication of liquid-metal-based conductors Three representative approaches: (A) Microfluidic elastomers. Reproduced with Permission (Gao et al., 2017). Copyright, Wiley-VCH. (B) Biphasic liquid metal alloy. Reproduced with Permission (Tavakoli et al., 2018). Copyright, Wiley-VCH. (C) Liquid-metal-embedded elastomers (LMEE) with self-healing property. Reproduced with Permission (Markvicka et al., 2018). Copyright, Springer Natur (D) Printable liquid metal-polymer LMEE. Reproduced with Permission (Tang et al., 2018). Copyright, Cell Press.

Tremendous different approaches to fabricate LM-based conductor and materials have been reported recently, making advantages and options out of its unique rheological property. Among all these, three representative strategies for LM-based conductor can be categorized into i) microfluidic elastomers, ii) biphasic alloys and iii) LM-embedded elastomers (LMEEs). All these strategies are discussed and compared in the following section (Figure 2).

## Microfluidic elastomer conductor

Microfluidic elastomers have been the most universal technique of using LM alloy for electronic sensors and circuit components with many other approaches including injection, direct writing, and contact printing (Figure 2A).(Khoshmanesh et al., 2017; Neumann and Dickey, 2020; Silva et al., 2020a) Unlike other solid materials, LM remain in liquid state without altering directly written traces or even can be injected into the silicones in a confined manner to form microfluidic elastomers. The microfluidic elastomers without any LM leakage typically exhibit highly consistent electromechanical characteristic (Khoshmanesh et al., 2017; Marques et al., 2019). However, all of these structures no matter how LM is deposited on to the donor substrate, they all require encapsulation layer to passivate the wiring structure. This also leads to low vapor diffusion rate at the interface between skin and the electronics that would cause irritations and other side effects. Thus, there are still persistent efforts in making high-resolution traces through high-vacuum-assisted filling or micro-nano-structure stamping for microfluidic elastomers for improvising patterning resolutions (Gao et al., 2017; Kim et al., 2020d). In addition, highly permeable fiber mats incorporating printed LM traces are also developed to prevent inflammation or irritation in further advancement in LM wearable electronics.

## Biphasic LM alloy conductor

Biphasic liquid metal alloys, a combination of a predeposited metallic either thin film or particles layer with face centered cubic (FCC) structure, such as Au, Ag, Cu, and Pt, for a donor layer and gallium-based alloys (eutectic gallium indium or Galinstan) for liquid wiring layer have been studied in recent times (Figure 2B).(Hirsch et al., 2016; Liu et al., 2021; Lopes et al., 2021) LM alloys can be easily alloyed onto the donor metallic layer agitating in a base environment or squeezing/tapping on the top of the layer, further enhancing electrical conductivity and stretchability. This strategy is typically advantageous with high-resolution patterning because the metal traces can be photolithographically defined or laser-micromachined in submicron scales, which is the smallest among the strategies. Nowadays, many recent progresses suggest that three-dimensional architecture in biphasic form can offer not only higher mechanical stretchability and electrical conductivity but also the other interesting properties (Ford et al., 2020; Liu et al., 2021; Lopes et al., 2021). Such approach incorporates suspended or precipitated heterogeneous metallic or nonmetallic materials where LM acts as a solvent or percolating interconnect. This approach could avoid applying applied strain to make electrically conductive percolating pathways within a composite of polymeric matrices. Moreover, there are many interesting versatile properties of these new approaches that could further be explored for LM-based stretchable and wearable electronics (de Castro et al., 2017; Kamysbayev et al., 2019; Tang et al., 2017; Wang et al., 2021; Wang et al., 2018).

## LMEE conductor

LMEEs are a composite of elastomers and LM alloy (Figures 2C and 2D). Once the gallium-based LM alloy undergoes shear mixing in prepolymer matrix or sonicated in alcohols, the thin native oxide on LM is formed and works as a surfactant, thereby remaining in droplet structures (Chen et al., 2020a; Daeneke et al., 2018; Lear et al., 2017; Lin et al., 2020; Zhang et al., 2019). With the theoretical yield stress of 200 MPa of the gallium oxide skin on the droplets (Tang et al., 2018), they do not easily bond back to bulk LM when agitated in a solvent. However, the oxide layer is broken easily under applied strain, tensile strain, or even peeling so that electrical connection can be made autonomously (Liu et al., 2019b; Tang et al., 2018). The LMEE introduced by Markvicka et al. can either be screen printed in a large area in a prepolymer state to create a soft elastic conductor by applying a strain afterward or mechanically plot the materials surface to rupture the surface to form on-demand connections (Figure 2C).(Markvicka et al., 2018; Neumann et al., 2020) The material also has electrically self-healing capability by forming new connections with neighboring LM droplets without interruption. However, for some of the polymer matrix or depending on LM volumetric percentages, the liquid droplets are suspended tightly in the matrix, making it useful for applications in mechanically compliant and metal-like material properties without electrical conductivity (Bartlett et al., 2017; Malakooti et al., 2019; Pan et al., 2019). Stretchable LMEEs can be printed and biocompatible by casting and peeling off polymers from prepatterned LM particles and confining metal layer inside polymeric matrix (Figure 2D).(Tang et al., 2018) These LMEEs have found useful applications for circuits, sensors, and functional wearables (Bartlett et al., 2017; Malakooti et al., 2019; Tang et al., 2018, 2021; Yao et al., 2020; Zadan et al., 2020). Table 1 is organized to show the materials properties of conductors in different LM architecture.

**Table 1:** LM-based stretchable conductors from different architectures and their categorized applications

LM architecture	Materials	Substrate	Stretchability	Electrical conductivity/resistivity	Applications	Reference
Microfluidics	Galinstan	Geniomer fibers	∼560%	3.46 × 10^6^ S m^−1^	Sensors, functional wearables	(Dong et al., 2020)
Microfluidics	EGaIn^a^	PDMS^b^ channel	∼180%	29.4 × 10^−6^ Ω cm	Sensors	(Jin et al., 2019)
LMEE^c^	EGaIn	3D Ecoflex	∼400%	1.1 × 10^6^ S m^−1^	Functional wearables	(Yao et al., 2020)
LMEE	EGaIn	PDMS matrix	∼50%	1.37 × 10^5^ S m^−1^	Circuits, functional wearables	(Markvicka et al., 2018; Zadan et al., 2020)
LMEE	EGaIn	PLC^d^	∼620%	8 × 10^3^ S cm^−1^	Circuits	(Cheng et al., 2020)
LMEE	EGaIn	PDMS	∼500%	7.02 × 10^5^ S m^−1^	Sensors, circuits, functional wearables	(Tang et al., 2018)
Biphasic alloy/LMEE	Ag^e^ flakes, EGaIn, SIS^f^	Elastomeric substrates	∼600%	7.02 × 10^5^ S m^−1^	Sensors, circuits	(Lopes et al., 2021)
Biphasic alloy	EGaIn, thin-film Cu^g^	PDMS	∼100%	1.77 × 10^−6^ Ω m	Circuits	(Pan et al., 2018)
Biphasic alloy	Ag ink, EGaIn	Tattoo paper	∼80%	4.85 × 10^6^ S m^−1^	Circuits	(Tavakoli et al., 2018)
Biphasic	EGaIn, Au	PDMS	∼30%	–	Circuits	(Kim et al., 2020d)

a EGaIn – Eutectic gallium indium.

b PDMS – Polydimethylsiloxane.

c LMEE – Liquid metal embedded elastomers.

d PLC – Poly(L-lactide-co-e-caprolactone).

e Ag – Silver.

f SIS – styrene-isoprene block copolymers.

g Cu – copper.

## Materials properties for LM-incorporated materials

**Figure 3:**
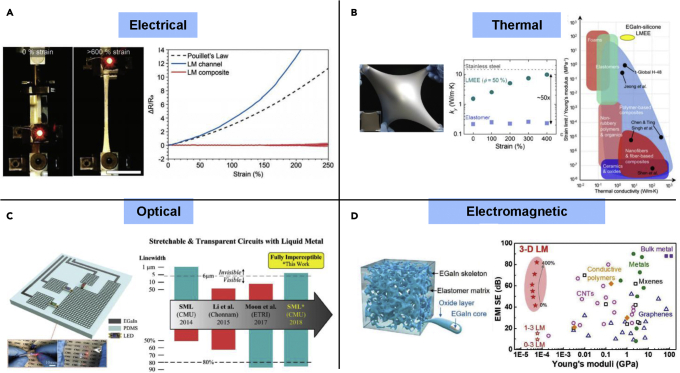
Unique properties exhibited from materials with liquid metal incorporation (A) High electrical properties insensitive to mechanical coupling of applied strains. Reproduced with Permission (Ford et al., 2020). Copyright, Wiley-VCH. (B) Soft rubbery material with metal-like thermal conductivity. Reproduced with Permission (Bartlett et al., 2017). Copyright, Proceedings of National Academy of Science. (C) Micropatterned high-resolution grid for high optical transparency. Reproduced with Permission (Pan et al., 2018). Copyright, Wiley-VCH. (D) Electromagnetic property of three-dimensional electrically conductive liquid metal composite. Reproduced with Permission (Yao et al., 2020). Copyright, Wiley-VCH.

Unlike solid metals, there are unique mechanical, electrical, thermal, optical, dielectric, and electromagnetic properties for LM-based materials (Figure 3). Among those, we will introduce the materials properties that are closely related to wearable electronic applications and discuss in the following text.

### Electrical conductivity

Electrical conductivity is a core value of determining conductor for sensors and circuits. Most gallium-based LM alloy (especially EGaIn and Galinstan [GaInSn]) or a composite are within a range of 10^5^–10^6^ S/m (Lear et al., 2017; Lopes et al., 2021; Mishra et al., 2020; Pan et al., 2018). Besides the LM channel without structural coupling from heterogeneous materials, there is minimized electromechanical in LM-based polymer 3-dimensional (3D) composites compared with linearly responsive coupling expected from Pouillet's Law (Figure 3A).(Fassler and Majidi, 2015; Ford et al., 2020) This originates from morphological effect in 3D percolating network of a composite (Zolfaghari et al., 2020). Because of this characteristic, the stretchable circuit made out of this structure will be highly desirable to strain-insensitive wearable electronics.

### Thermal conductivity

Because most elastic materials for wearables show low thermal conductivity while rigid metals exhibit orders of magnitude higher, there has been a huge trade-off in thermal and mechanical properties in wearable electronic materials (Lee et al., 2020b; Zhan et al., 2018). Bartlett et al. have introduced that LM droplet inclusion with an insulation polymer realizes the material with both high thermal conductivity (4.7 ± 0.2 W·m−1 ·K−1, when strained 9.8 ± 0.8 W·m−1 ·K−1) and low modulus (∼100 kPa) and stretchability (<700%) (Figure 3B).(Bartlett et al., 2017) This is a very astonishing result that advances low thermal conductivity of polymer (0.1–0.2 W·m−1 ·K−1) (Hou et al., 2019) while minimizing the loss in thermal conductivity of pure LM (48 W·m−1 ·K−1) (Je and Lee, 2014). Therefore, this thermally conductive rubbery material has possibilities to offer rapid heat dissipation or delivery for future thermal wearable applications.

### Optical transparency

Transparency can be an important feature in wearable electronics by providing unobtrusive wearability and see-thorough functionality (Melzer et al., 2015; Takemoto et al., 2020; Won et al., 2019, 2020). Transparent electrodes with high optical transparency, electrical conductivity, and flexibility have already been extensively investigated for optoelectronic devices and transparent wearable electronics (Kwon et al., 2018; Sannicolo et al., 2016; Tan et al., 2017). However, most of transparent conductors that high hysteresis and experiences high ratio trade-off between optical transparency and electrical conductivity.

To address current challenges for transparent electrodes, Pan et al. have used a biphasic conductor, where a copper thin film (100 nm) is alloyed with LM and combined with laser-based microfabrication technique (Figure 3C).(Pan et al., 2018) Owing to strong metallic bonding between solid copper support layer and upper LM conductive layer, it allows to resist residual stress during ablation patterning process and yielding the finest line width among LM traces as small as 4.5 μm and pitch of 100 μm. It has shown to produce optically transparent (<85% at 550-nm wavelength) electrodes (with R_sheet_ = 2.95 Ω/□) that ensure a strain up to 100%. Owing to such its optical property and mechanical deformability, LM-based wearable electrodes will have a broad range of applicable areas from wearable circuits and sensors to wearable display and see-through bioelectric monitoring devices (Lee et al., 2017; Yokota et al., 2016, 2021).

### Electromagnetic property

Electromagnetic interference (EMI) shielding is becoming necessary as an increased number of wearable personal electronic devices may fall in a danger of information leakage and false operations or even human health from long-time exposure to electromagnetic radiation (Jung et al., 2017; Li et al., 2020a). Two representative barometers for EMI shielding are reflection and absorption, which arise from direct interaction of mobile charge carriers and electric/magnetic dipoles in shielding materials with the EM fields accordingly. The electrical conductivity of the materials is known to be the most influential parameter in determining the EMI shielding effectiveness. However, a number of efforts for making EMI shielding composites with conductive fillers up to date still experience a loss in shielding effectiveness owing to decreased conductivity under constant deformation (Gupta et al., 2020; Jung et al., 2017; Li et al., 2020a; Pu et al., 2018).

Yao et al. has found out that an LMEE in a 3D network can maintain electrical conductivity that is closely similar to the EMI shielding effectiveness of metallic plates for a broad range of 2.65–40 GHz (Figure 3D).(Yao et al., 2020) Therefore, such a composite in 3D network with high mechanical compliance could potentially provide a route for new functionalities in soft wearable electronics and materials.

## Recent advances in LM-based wearable electronics

Many wearable sensors are recently developed based on LM as either sensory layer or circuits owing to its high electromechanical stability and linearly mechanically coupled response. The LM-based sensors with strain/pressure tactile sensing wearables, electrochemical sensors, and haptic devices are reviewed in this section.

### Tactile wearable sensors

**Figure 4:**
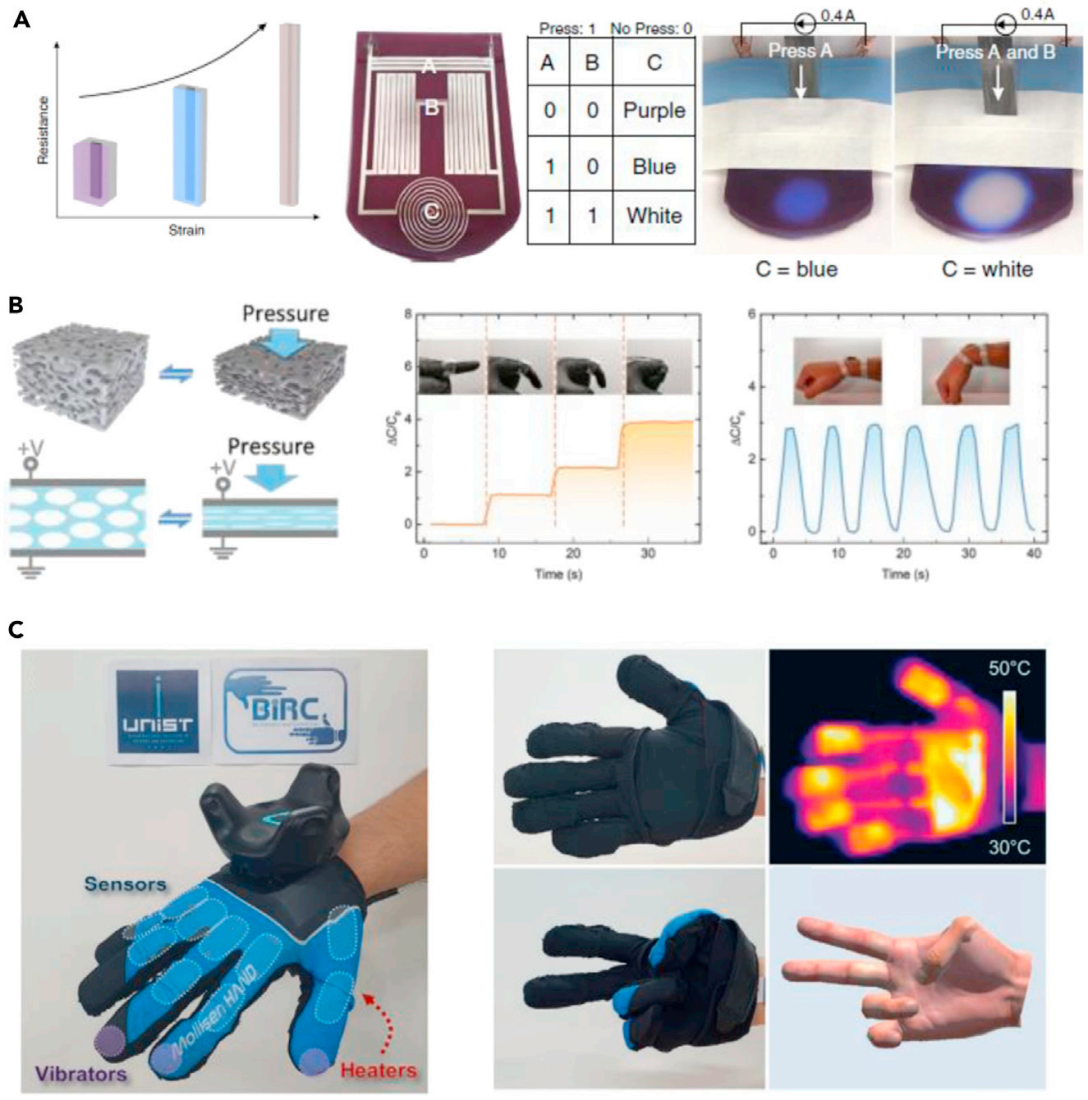
Liquid-metal-based wearable tactile sensing element (A) Soft tactile logic sensor. Reproduced with Permission (Jin et al., 2019). Copyright, Springer Nature. (B) Soft porous foam as a capacitive tactile sensor. Reproduced with Permission (Yang et al., 2020). Copyright, Wiley-VCH. (C) Multimodal haptic glove with thermal and tactile sensation. Reproduced with Permission (Oh et al., 2020). Copyright, Wiley-VCH.

Although LM-based soft tactile sensors usually exhibit low gauge factor (>10) in measuring strain and pressure, an accurate linear response of liquid metal channel can be used to express mechanical deformation precisely (Kim et al., 2018; Park et al., 2012). In a recent work by Jin et al., the microfluidic elastomer filled with LM and covered with thermochromic layer uses its deformable ability to couple the changes in Joule heating to thermochromic and mechanochromic sensing of touch and strain (Figure 4A).(Jin et al., 2019) Basically, when this device is being stretched or pressed, there is the changes the cross-sectional space of LM and therefore changes the resistance. Consequently, the device works as a tactile sensor by delivering the stress to the change in colors. Finally, the proposed device also has demonstrated soft tactile logic that makes own decisions to respond locally to environmental interactions or act as embedded sensors for feedback loops. Because of these capabilities, such tactile logic sensors can potentially be served as wearable electronic skin or electronics for prosthesis and more accurate human-machine interface.

Capacitive sensors made of electrolyte fluids, conductive polymers, and metallic nanomaterials have been studied for stretchable tactile sensing component (Cai et al., 2013; Kalantar-Zadeh et al., 2019; Kang et al., 2017; Kim et al., 2017). In comparison with resistive sensors (Kim et al., 2019b; Kwon et al., 2016; Lee et al., 2012a), the capacitive tactile sensors usually sustain the original performance as long as there is a soft insulation layer to avoid direct contact between two capacitive electrodes upon repetitive mechanical deformation or pressure (Park et al., 2015). Pressing the foam closes the gap between the electrodes and thereby increases capacitance. Because the sensitivity to a given force is maximized by using soft dielectric materials with higher dielectric constant, Yang et al. have fabricated an LM-embedded porous foam to demonstrate soft, compressible, and high dielectric constant (Figure 4B).(Yang et al., 2020) Compressing the LMEF displaces the air in the foam structure, maneuvering the permittivity over a large range (5.6–11.7), but at larger strain, it starts to behave the other way around. The change in permittivity allows using this material as a soft wearable tactile sensor with high sensitivity, high initial capacitance, and large capacitance change.

VR can be realized with tactile sensation, which not only senses the change in motion but also gives stimulus to the wearable users. This system is so called, “haptics,” that realizes tactile sensing and tracking motion activity. This stand-alone platform enables a contact-free technology in a virtual world where gaming, training, and entertaining by himself/herself is possible in a forthcoming area (Kim et al., 2020a, 2020b; Lee et al., 2020a, 2020b; Mishra et al., 2020; Park et al., 2020). Oh et al. have enabled this technology using a multimodal sensing and feedback glove consisting of stretchable sensor and heater layer manufactured by direct ink writing of LM (Figure 4C). The ten strain sensors on fingers and three vibro-haptic vibrators integrated with multilayered heaters provides thermo-vibro haptic sensation in accurate and rapid manner based on feedback control even under stretched conditions. The performance of this wearable tactile sensing multimodal glove is verified in the VR world by gasping, touching, and pushing different materials at different temperature regimes for wearable haptic applications.

### Various other LM-based wearable sensors

**Figure 5:**
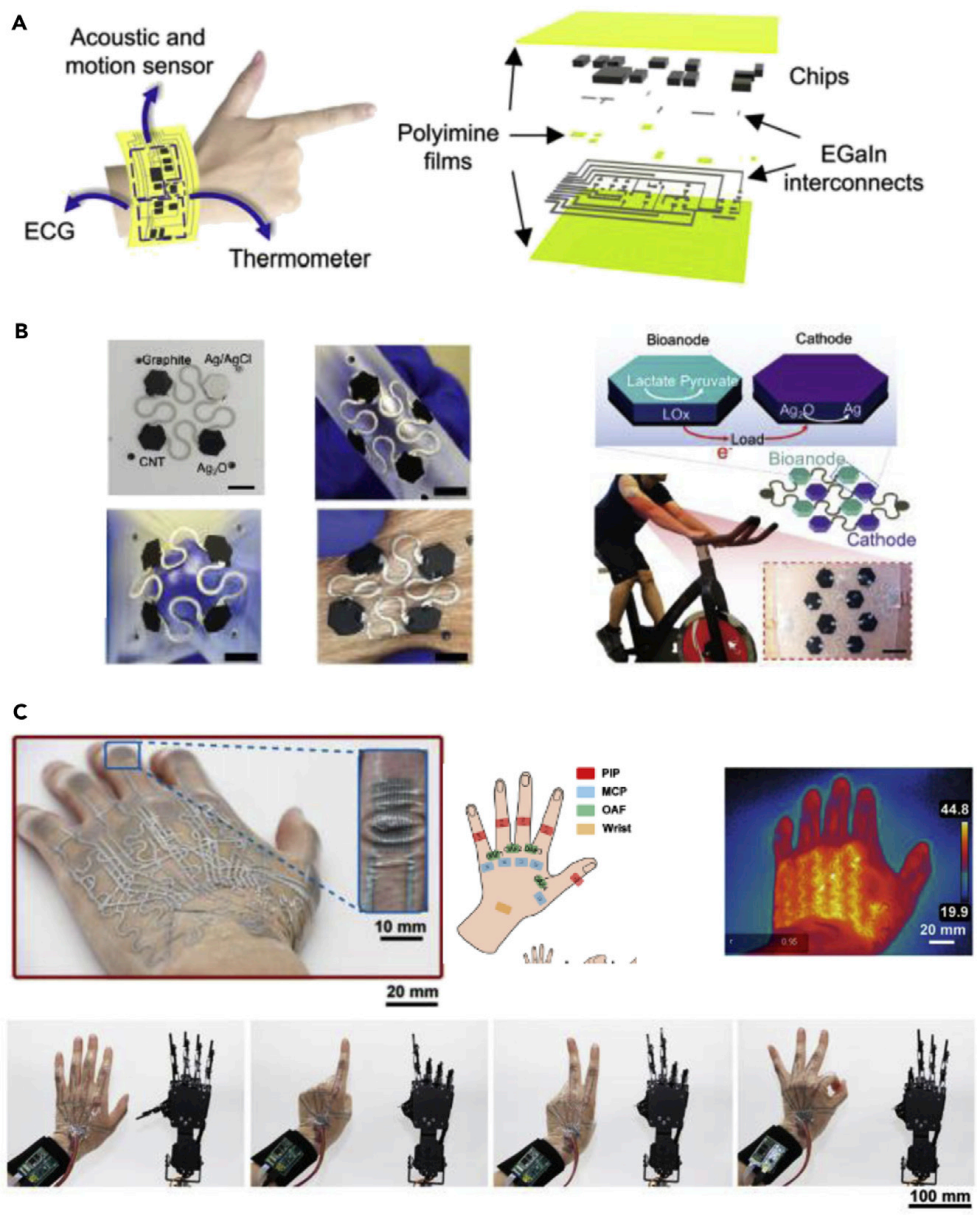
Various other liquid-metal-based wearable sensors for sensing, monitoring, and machine-interaction applications (A) Heterogeneous integration of polymer and liquid metal for mechanically stable multifunctional wearable electronics with acoustic, motion, electrocardiogram, and temperature monitoring capabilities. Reproduced with Permission (Shi et al., 2020). Copyright, AAAS. (B) Wearable electrochemical sensor composed of island and bridge architecture. Reproduced with Permission (Silva et al., 2020b). Copyright, Wiley-VCH. (C) Multilayered electronic transfer tattoo (METT) for human machine interaction or haptic applications using the crease amplification effect. Reproduced with Permission (Tang et al., 2021). Copyright, AAAS.

Several other applications well-suited for skin or body interfaced sensors have been recently proposed. Shi et al. have demonstrated multifunctional sensor arrays that provide motion tracking, temperature monitoring, and sensing of acoustic vibrations and electrocardiogram signals (Figure 5A).(Shi et al., 2020) The LM circuitry encapsulated with a covalent polyimine layer realizes highly stretchable, self-healable, recyclable, and reconfigurable characteristics (Liao et al., 2019; Teng et al., 2019a). This is enabled by the bond exchange reactions in the polyimine network and fluid behavior of the LM circuitry. The multifunctional components that this device is capable of can be used for continuous wearable monitoring, prosthesis, and therapeutics. In contrast to other electric systems, the recyclability into the monomers, microchips, and LM droplets by dissolving them in solvents, when this device is severely damaged or no longer functional, is particularly noble, thereby ensuring sustainable economics from such electronics. Furthermore, patterning of these LM circuitry in serpentine (Yeo et al., 2013) or open-mesh (Lee et al., 2017) structure may advance in mechanical compliance, skin breathability, and other biomedical imaging compatibility for wearable bioelectronic applications (Jang et al., 2014; Kim et al., 2011).

Extraction of chemical fluids from the body, for monitoring health conditions and harvesting energy, has taken substantial attraction for wearable chemical sensors (Bandodkar et al., 2019; Chen et al., 2019; Choi et al., 2018; Jeerapan et al., 2020; Koh et al., 2016). Silva et al. introduces an island-bridge (IB) architecture that is composed of silver (Ag) and eutectic gallium indium particles (EGaInPs) serpentine bridges to enable the printed microstructures to maintain mechanical and electrical properties under an extreme (≈800%) strain (Figure 5B).(Silva et al., 2020b) By integrating the bridges with a separate sensing element, the rigid island electrodes printed with carbon allow to detect electrochemical responses and sustain high mechanical deformation while capturing stable electrochemical analytes. In addition, the printed IBs can use biofluids collected from human sweat as a power source for wearable electronics. Hence, the electrochemical sensor presented here may well suited for a wider range of applications in wearable electronics toward sensing and harvesting.

Conformability of wearable electronics can make a big difference owing to the crease amplification effect as studied by Tang et al. (Kim et al., 2020c; Tang et al., 2021; Yeo et al., 2013) The crease amplification effect means that conformal interface and bonding between sensing layer and measuring surfaces such as the skin with curvilinear surfaces will lead to amplification of output signals to respect the changes in strain. A layer-by-layer fabrication method that is used here creates multilayered electronic transfer tattoos (METT) while retaining the crease amplification effect. Using the three-layered METT that covers the entire hand for 15 degree of freedoms, the METT can be integrated with the robotic control system to demonstrate precisely controlled human-machine interaction, which would have a great potential in the medical surgery system, VR, and several human-machine interfaces (Figure 5C) (Choi et al., 2017; Diana and Marescaux, 2015; Sim et al., 2019).

## LM-based energy-harvesting and storage devices

There has been an increasing demand for portable wearable energy system that can store and harvest for a prolonged usage of personal electronics (Song et al., 2020a; Xie and Wei, 2014; Xu et al., 2013). In this section, we introduce the development of soft wearable energy-harvesting devices and storage devices assisted by LM incorporation.

### Triboelectric nanogenerator

**Figure 6:**
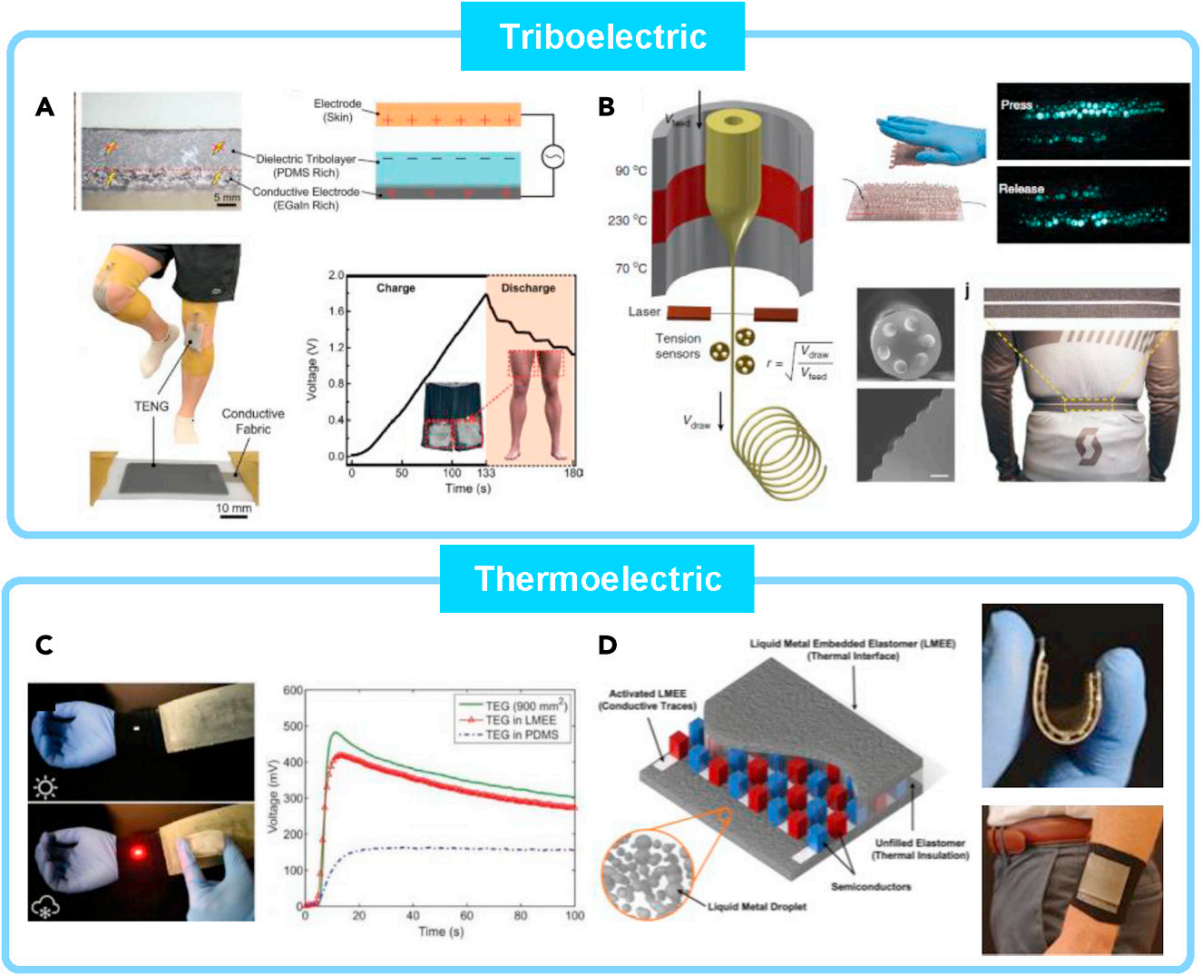
Liquid-metal-based energy harvesting systems using wearable triboelectric nanogenerators (TENG) and thermoelectric generators (TEG) (A) LMEE-coated textile for TENG interfaced naturally with a skin. Reproduced with Permission (Pan et al., 2020). Copyright, Wiley-VCH. (B) Thermally drawn elastic fibers integrated with liquid metal as TENG device electrodes for smart textiles, having sensing and energy-harvesting capabilities. Reproduced with Permission (Dong et al., 2020). Copyright, Springer Nature. (C) Thermoelectric generator using supercooling effect of liquid metal. Reproduced with Permission (Malakooti et al., 2019). Copyright, Wiley-VCH. (D) Liquid metal embedded elastomers for both circuits and thermally conductive layers. Reproduced with Permission (Zadan et al., 2020). Copyright, American Chemical Society.

Energy-harvesting wearable devices that can convert large-scale mechanical motions into electrical power have been proposed as one of the ways to solve the problems in power devices. Triboelectric nanogenerators (TENGs) have attracted attention especially for LM-based wearable TENGs because of its notable robustness and low electromechanical hysteresis, allowing its abrasion resistance to repeated cycles of motions (Figure 6) (Tang et al., 2015; Teng et al., 2019b; Yang et al., 2018).

Soft and stretchable TENGs are demonstrated with LMEEs (Figure 6A). Pan et al. have made LMEEs that are intentionally sedimented LM (SLM) droplets for TENG energy-harvesting layer that requires both insulating and conductive sides (Pan et al., 2020). The SLM elastomer TENG (SLM-TENG) demonstrates high stretchability, mechanical compliance, and electromechanical stability for 10,000 cycles and moderate electrical output performance (the maximum power density = 1 mW cm−2). SLM-TENG layers can be easily coated on a several stretchable fabrics, enabling broad integration with wearable electronics. Hence, SLM-TENG is demonstrated that harvesting energy from human motion from a patch attached to the knee part during the exercise. It has demonstrated that the mounted TENG device can generate enough electricity to fully power a wearable digital hydro thermometer after a few min of running. These demonstrations suggest that LM-based wearable TENG devices are highly resilient and abrasion-resistant under dynamic motions and movements.

Fibers are suitable form of a material that harvest mechanical energy by the triboelectric effect and power sources for wearable electronics and functional textiles. Dong et al. have demonstrated the scalable fabrication of microstructured stretchable triboelectric fibers (Figure 6B) (Dong et al., 2020). The team uses the thermally drawn elastomeric fibers that are extruded from predefined microtextured Geniomer granules and several lines of LM electrodes are injected inside later. The fibers show high electrical conductivity regardless of repeated cycles of large deformations and have stretchability up to 560%. They are sewable to woven textiles with high electrical outputs up to 490 V, 175 nC by triboelectrification. These electronic fibers also can be demonstrated with self-powered breathing monitoring system and gesture sensing feature, using the fiber for both sensing and energy-harvesting elements for wearable electronics and smart textiles.

### Thermoelectric generator

For most wearables, it is very natural that there are temperature differences between the inside that interfaces with body and the outside that is exposed to the surrounding condition. Consequently, to make use of the heat waste coming from the body, thermoelectric wearables have drawn much attention recently, but there are two major requirements: i) soft compliant thermally conductive layer and ii) soft circuitry array because a conventional thermoelectric generator has brittle and rigid thermally conductive ceramics or metals at the top and bottom and a circuit that is not stretchable (Hong et al., 2019; Lee et al., 2020b; Oh et al., 2016; Ren et al., 2021; Suarez et al., 2017; Sun et al., 2020a).

At first, Malakooti et al. have used aforementioned LMEEs made of Ecoflex and EGaIn as a thermally conductive, mechanically compliant layers to conventional thermoelectric generator to show an efficient heat extraction for energy conversion (Figure 6C) (Malakooti et al., 2019). By controlling the size of LM droplets (∼3 μm) and selection of polymeric matrix, the layer can be applied in a wide temperature ranges (freezing point down to −84.1 from −5.9°C) that other fluidic system could not. Moreover, the same LMEE but with PDMS not only exhibits high thermal conductivity but high electrical conductivity only on the mechanically scribed surfaces (Figure 6D) (Zadan et al., 2020). The LMEE composites are then used for both thermally conductive layers and circuits. With integrated array of Bi2Te3 semiconductor micropallets, these stretchable wearable LMEE thermoelectric devices can generate voltages of 59.96 mV at Δ10°C, 130 mV at Δ30°C, and 278.6 mV and be stretched greater than 50%. Therefore, these thermoelectric devices suggest a good alternative to energy-harvesting generator especially for wearables (Zadan et al., 2021).

### Battery

For an efficient energy storage device for portable and wearable electronics, current wearable electronic market uses high power density with lighter or miniaturized energy storage devices such as Li-ion batteries for smart watches, glasses, and other wearable sensors (Liu et al., 2018; Zhang et al., 2016). However, there are two largely affecting limitations, which impede the scope of wearable battery as well as the performance of electronics, in integrating these batteries in a fully soft and stretchable manner to humans. i) Hard and rigid nature of metal-plated cathode and anode and ii) interstitial impedance between soft and hard materials would cause explosion/degradation by unnecessary dendrite growth (Liu et al., 2018). Nevertheless, a lot of efforts to fabricate batteries more flexible and stretchable has been carried out through engineering architecture and materials development for electrodes, electrolytes, and separators that comprise a battery architecture without electromechanical, chemical, and safety issues under deformations.

**Figure 7:**
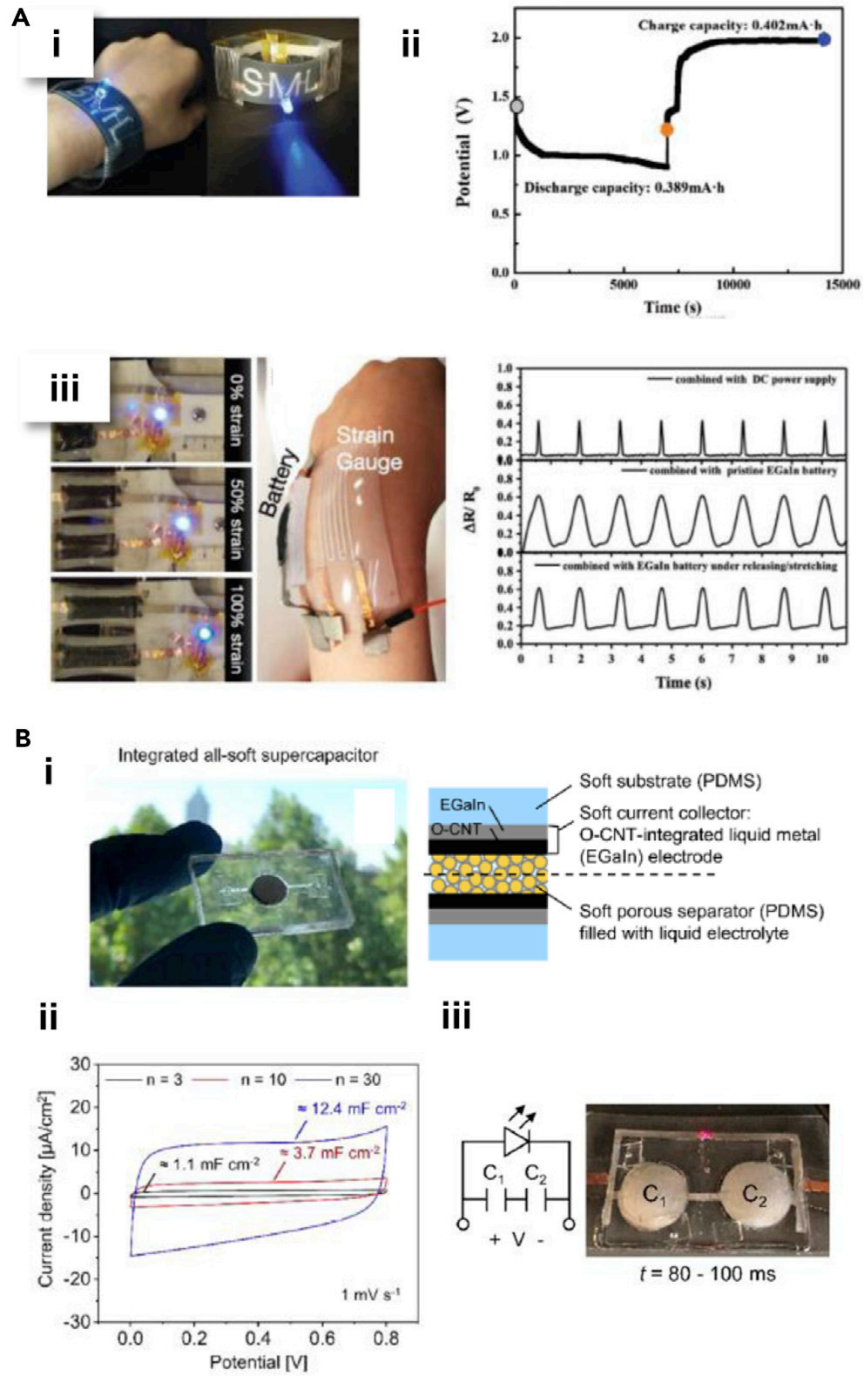
Liquid metal-based wearable energy storage devices (A) soft stretchable EGaIn-MnO2 battery.(A,i) Demonstration of lighting a blue light-emitting diode (LED) using soft wearable battery in a form of a wearable sleeve. (A,ii) charge and discharge curves of the soft battery. (A,iii) Soft wearable strain gauge powered by a wearable battery. Reproduced with Permission (Liu et al., 2019a). Copyright, Wiley-VCH. (B) All-soft supercapacitors with EGaIn electrodes and oxidized carbon nanotube (O-CNT). (B,i) A captured image of integrated all-soft supercapacitor with description of architecture on the right. (B,ii) Voltammetry cycle respect to number of cells tested. (B,iii) Reproduced with Permission (Kim et al., 2020e). Copyright, American Chemical Society.

To tackle aforementioned issues, all soft stretchable battery is proposed by Liu et al. to demonstrate that an EGaIn LM alloy can be the solution by using it as an anode while having an MnO_2_ cathode as a counterpart cathode (Figures 7Ai-ii) (Liu et al., 2019a; Zeng et al., 2017). As a simple proof of a concept, a fluidic electrode such as LM alloy can address such challenges by cracks and dendrite growth that other interfacial problems are suffered from a rigid electrode. The cathode is also made to be stretchable by prestretching a composite of carbon paste and MnO2 slurry and embedding within elastomeric matrix to form a wavy film that mitigate the stress under mechanical deformations. Therefore, all soft battery architecture is constructed with a LM alloy as an anode, a carbon paste composite slurry with MnO2 as a cathode and two different anodic/cathodic alkaline hydrogel electrolytes that are doped with alkaline KOH only and LiOH/KOH ions, respectively, in PAAm matrices. Moreover, integration of CaCl2 additives to EGaIn has increased effective contact area between anodic hydrogel and stabilizes electrochemically for better battery performance. Because of deformability of these soft materials altogether, the battery has also shown highly stabile rechargeability for 100 cycles at 0.4 mA cm^−2^ and 60 cycles at 1 mA cm^−2^, both under bending and tensile strains. The EGaIn-MnO2 battery is then fabricated into elastomeric wearable sleeve so that it can provide power to a blue light-emitting diode (LED) and a strain sensor (Figures 7Aiii). Such an example of a soft wearable battery has recently been pursued that LM will play a critical role in wearable energy storage devices to solve problems in accommodating human motions.

### Supercapacitor

Supercapacitors is another form of energy storage device that shows fast charging/discharging rate, high power and energy densities, long-term cycling, and safe operation (Borenstein et al., 2017; Lee et al., 2015, 2016; Moon et al., 2017; Najib and Erdem, 2019). A supercapacitor consists of two electrodes that sandwiches an electrolyte in between them. Most supercapacitors that are proposed so far either flexible or geometrically engineered stretchable form to realize wearable storage system. The prerequisites for high-performance wearable supercapacitors typically require i) mechanical deformability and stability of an electrode, ii) large surface area of an electrode for efficient ion transport with an electrolyte, and iii) electrochemical stability of capacitive electrodes (An and Cheng, 2018; Chen et al., 2020b).

With an aid of LM alloy, Kim et have demonstrated that all soft supercapacitors can be constructed by synergistically combining it with an oxygen functionalized carbon nanotube (O-CNT) (Kim et al., 2020e). The EGaIn (≈1.5 μm) as an LM electrode is stamped onto paper-textured PDMS substrate layer first to serve as a support electrode for O-CNT electrode and form a strong bonding with its native oxide (Figure 7Bi) (Kim et al., 2020e). Thus, this proposed architecture can withstand applied strains without delamination between high conductive electrical pathways and 3D CNT network. Although O-CNT can generate cracks under tensile strain, the EGaIn support beneath the layer heals the gap and brings it reversibly back to original state both mechanically and electrically owing to the strong bond. Along with these vertically aligned electrodes, a soft porous PDMS separator filled with ionic liquid electrolyte is placed in the middle to complete all soft supercapacitors. The fabricated all soft supercapacitors have shown stable electrochemical and electrotechnical properties under deformations while exhibiting areal capacitances as high as 12.4 mF cm^−2^ and unchanged performance up to 30% tensile strain (Figures 7Bii). They also maintain >95% of their original capacitance after >4200 charging and discharging cycles with a periodic applied strain of 30%. Finally, all soft supercapacitors integrated with a commercial LED have shown to integrated soft wearable system (Figures 7Biii).

## Wireless energy harvesting and communication

Soft wearable antenna is an essential component of wearable electronics for portable wireless operation, wireless energy harvesting, and communication. Because the inductive coil for the antenna determines resonance frequency as per resistance, inductance, and capacitance circuit that matches with desired frequency ranges, its circuit crucially requires good patternability, electromechanical property with low gauge factor, and hysteresis (Abdelraheem et al., 2019; Heo et al., 2018; Kim et al., 2015a, 2015b).

**Figure 8:**
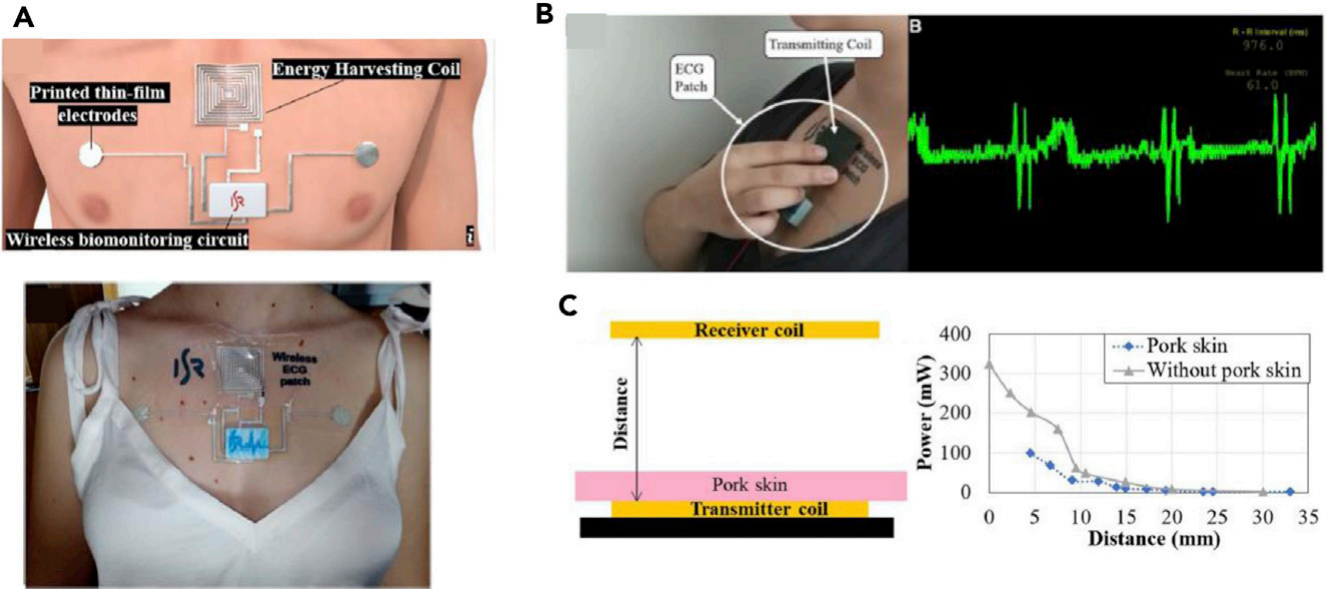
Liquid-metal-based wearable wireless power transmission and communication device (A) Schematic illustration of wireless energy harvesting coil and an actual image of integrated device onto the skin. (B) Demonstration of untethered monitoring of electrocardiogram (ECG) by using wireless passive wireless antenna. (C) Energy harvesting capability with/without pork skin depending on displace from transmitter coil. Reproduced with Permission (Alberto et al., 2020). Copyright, American Chemical Society.

Initial attempt to use LM in wireless wearable electronics is proposed by Alberto et al. to demonstrate an electronic tattoo that has functionalities such as battery-free wireless energy harvesting from passive antenna to monitor electrophysiological monitoring (Figure 8A) (Alberto et al., 2020). This tattoo pattern is printed on a tattoo paper from a desk top printer by loading the Ag flakes ink, and it is sintered by EGaIn LM alloy, making it highly conductive and stretchable. Inductive coil patterns together with skin-interfacing electrodes made in Ag-In-Ga enables wireless power transfer (WPT) health monitoring system (Figure 8B). It shows that the WPT can provide ∼300mW of power, which is within an operating range of biomedical devices (Figure 8C). This device aims to assist patients in and out of hospitals by simply sticking onto the skin and analyzed by doctors and nurses.

Similarly, a composite of Ag microflakes, LM microdroplets, and styrene-isoprene block copolymers, Lopes et al. has presented the digitally printed sensors, circuits, and antenna that are highly deformable and wearable (Lopes et al., 2021). This 3D-structured electronic material composite is very cohesively connected in biphasic form so that the printed ink shows stable electromechanical performance with a low gauge factor of 0.9 and a stretchability up to 600%. The major advantage of this trinary composite, where EGaIn, Ag flakes, and AgI_2_ microparticles play each role, is nonsmearing and nonmarking properties, which are unlike previous liquid-like biphasic thin-film structure particularly hinders integration of microchip components without encapsulation. More importantly, this fluid-like deformability and microchip integrity highly improve mechanical stability for sensors and circuits for wearable monitoring, wireless harvesting, and data communication. For better comparison, Table 2 is made to show the performance of all the energy-harvesting and storage devices with various properties including the power output performances.

**Table 2:** LM-based wearable energy harvesting and storage devices

Energy harvesting/storage devices	Materials	Substrate	Stretchability/Wearability	Power output performance	References
TENG^a^	Galinstan	Geniomer fibers	∼560%, 50,000 cycles (compression and stretching)	490 V, 175 nC	(Dong et al., 2020)
TENG	EGaIn^b^	Ecoflex matrix	∼500%/10,000 cycles (at strain of 100%)	∼1 mW cm−2	(Pan et al., 2020)
Battery	EGaIn	Ecoflex	∼100%	3.8 mA cm−2	(Liu et al., 2019a)
Supercapacitor	EGaIn, O-CNT^c^	PDMS^e^	∼50%/2000 cycles (at strain of 30%)	12.4 mF cm−2	(Kim et al., 2020e)
TEG^d^	EGaIn	Ecoflex	–	250–400 mV at Δ37°C	(Malakooti et al., 2019)
TEG	EGaIn	PDMS matrix	∼50%	59.96 mV at Δ10°C, 130 mV at Δ30°C, 278.6 mV, 86.6 μW/cm2 Δ60°C	(Zadan et al., 2020)
Wireless	Ag^f^ inks, EGaIn	Tattoo paper	–	300 mW in air, 100 mW with skin	(Alberto et al., 2020)

a TENG – Triboelectric nanogenerator.

b EGaIn – Eutectic gallium indium.

c O-CNT – Oxidized carbon nanotube.

d TEG – thermoelectric generator.

e PDMS – Polydimethylsiloxane.

f Ag – Silver.

## Implantable wearable bioelectronics

Tissue-like wearable bioelectronics can serve as therapeutics and continuous monitoring systems and even artificial replacements. For those who suffer from diseases or lost typical functionality of organs, researchers have come up with implantable wearable bioelectronics that matches with its original functionality or even assist therapeutically (Li et al., 2021; Scholten and Meng, 2015).

**Figure 9:**
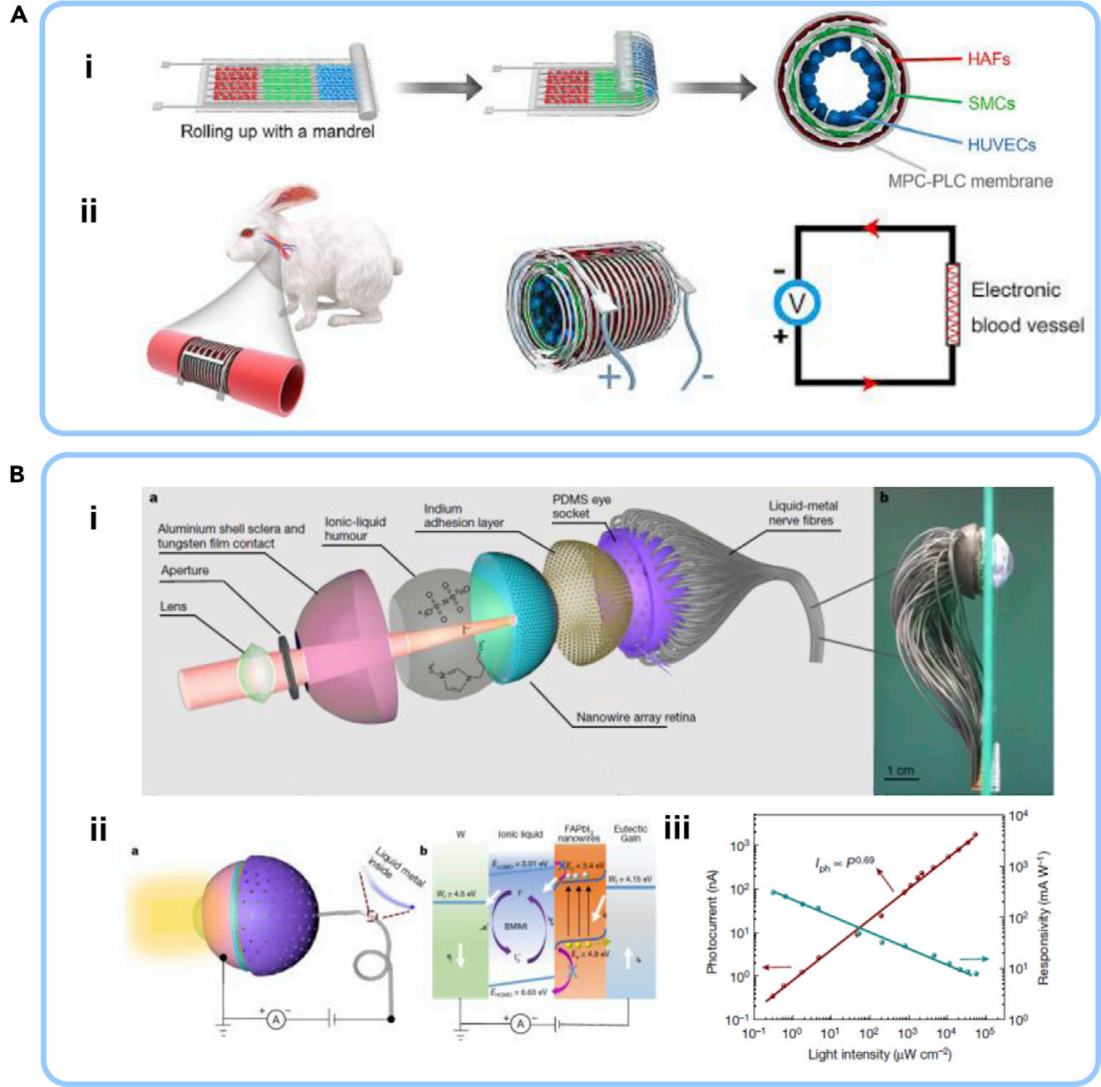
LM enabled implantable wearable bioelectronics (A) Electronic blood vessel. Reproduced with Permission(Cheng et al., 2020). Copyright, Cell Press. (A,i) Schematically illustrated architecture of electronic blood vessel. (A,ii) Application of electronic blood vessel to a rabbit model to demonstrate improved therapeutics thorough electrically stimulated *in situ* endothelialization process and electroporation. (B) A biomimetic artificial eye. (B,i) An artificial eye architecture wiring with liquid metal fibers as electrical nerves. (B,ii) illustration of photodetection from light excitation to the artificial eye. (B,iii) The photocurrent response to the artificial eye respect to the illuminated light intensities. Reproduced with Permission (Gu et al., 2020). Copyright, Springer Nature.

Recent study from Cheng et al. has looked at cardiovascular diseases, which is the number one cause of the mortality, for developing an implantable “electronic blood vessel” (Cheng et al., 2020). During the surgery of cardiovascular diseases, there is a great demand for a small-diameter (<6 mm) tissue-engineered blood vessel that provides both a mechanical support and assists its regeneration by facilitating the endothelialization process of tissues after surgery (Seifu et al., 2013). For this reason, by fabricating LM-based circuits with a biodegradable polymer and growing three different cells, HAFs, SMCs and HUVECs on top of the circuitry, an electronic blood vessel, with excellent biocompatibility, electromechanical properties, and degradability, enables electrical stimulation to facilitate targeted *in situ* endothelialization process and electroporation to deliver desired genes among the grown cells to the vessels (Figure 9Ai). The electronic blood vessel remains safe and functional after 3 months of implantation in a rabbit model (Figure 9Aii). In the future, the electronic blood vessel can be integrated with other electronic components and devices to enable diagnostic and therapeutic functions and greatly empower personalized medicine by creating a direct link in the vascular tissue-machine interface.

Artificial organ is one of great interests in modern days in biomedical engineering for those people who need extra implantation or replacement to properly function in daily lives (Annesini et al., 2017). Gu et al. have proposed an artificial eye mimicking capability of human eyes that possess image wide-sensing range, high resolution, and sensitivity (Gu et al., 2020). These human-inspired eyes can be particularly desirable in robotics and visual prostheses (Mishra et al., 2020). Although making the retina's hemispherical shape similar to an actual eye has many technical challenges, the development of nanomaterials, microfabrication/nanofabrication technique and finally liquid-injected microcircuitry arrays has enabled the fabrication of electrochemical photodetectors array on a nonplanar substrate. The artificial eye structure is schematically illustrated in Figure 9Bi, where the band alignment depicts tungsten, ionic liquid, FAPbI3 perovskite layer, and EGaIn can lead to charge carrier separation under light excitation. It shows that the dependency of the photocurrent highly sensitive responds to the illuminated intensity (Figure 9Bi-ii). Not only mimicking the eye, this device will also further inspire approaches for optical imaging devices that can be applied in that could find application in implantable electronics and soft robotic eyes.

## Conclusion and outlook

In this report, we have reviewed recent advances in LM-based wearable electronics and materials, which typically yield opportunities created with different fabrication methods, materials properties, and applications. Benefits of their electrical, thermal, optical, and dielectric properties are well in accordance with various wearable electronic devices applications by using these material properties. There are three categories of making LM-based stretchable conductors – i) microfluidic elastomers, ii) biphasic LM alloy, and iii) LMEEs in which they can be synergistically combined to improve device performances or materials properties. Although their superior mechanical and electrical properties under deformations compared with patterned solid film and nanomaterials usually limit LM-based layer as circuits or strain sensor where only low gauge factor is needed, by integrating or mixing with heterogeneous conductive materials and polymeric matrices, many groups have recently created new applications in sensors, energy devices, antennas, and implantable devices. Although, there will be remaining challenges regarding fabrication processability, high-level integrated system, and device performance improvements for LM-based wearables and materials.

1) Most of LM-based stretchable conductors are highly processable and easy to scale-up at low cost. There are still opportunities if the LM-based circuits and sensors can readily be applied to the body-level-sized exoskeleton suit that can enable 3D monitoring/mapping of active motions of the entire body. At such larger scale applications, it can be used to manipulate the whole body in virtual reality no one ever has experienced before. We expect that this type of manufacturing system can be developed through printing or molding technologies using LM composite materials. Recent approach using supersaturated LM with other metal or nonmetal can be the key to define highly controlled structure in scalable manner or current approaches that only incorporate polymers and LMs could be enough.

2) Beside high stretchability and conductivity, LM-based patterns do not provide high sensitivity in many sensible areas. However, either by using stable electrical connections for stable sensing/heating performances or by integrating with new materials to present different or multifunctional sensing capabilities, the higher level of integrated sensors and device system as well as the performance improvements are two major approaches to find tremendous applications in human-friendly wearable devices. We strongly believe that more forthcoming advances in LM-related research will bring many changes to outfits and wearable electronics in everyday life.

3) Biocompatibility of LM-based electronics and materials need to be thoroughly validated and considered before developing all of aforementioned applications. Many metals could be cytotoxicity to the skin owing to allergic reaction or ion diffusion to skin cells. Although gallium-based LMs are comparably safer than mercury owing to low vapor pressure, recent study has shown that there is ion different from both Ga and In when a bulk EGaIn was placed in aqueous environment. This could imply that EGaIn would cause skin cells to die if it is not properly architecture. Therefore, it would be important to understand the mechanism behind the cytotoxicity of LM and proper employment of LM architecture should be applied for further advances in LM-based wearable technology.
